# The interface between paediatrics and camhs (child and adolescent psychiatry): Mental state examination teaching for paediatric trainees

**DOI:** 10.1192/j.eurpsy.2021.599

**Published:** 2021-08-13

**Authors:** G. Xu

**Affiliations:** Psychiatry, Central and North West London NHS Foundation Trust, London, United Kingdom

**Keywords:** mental state, Teaching, paediatrics, interface

## Abstract

**Introduction:**

During an out-of-hours shift, the initial assessment of a CAMHS patient is performed by the paediatric trainee, usually the paediatric SHO (senior-house-officer). During my placement as a paediatric SHO, I was aware of a gap in formalised metal state examination teaching for paediatric juniors, which would be crucial for a thorough assessment of these patients, and to better guarantee they are safely managed until further assessment.

**Objectives:**

The aim is to provide a short teaching session on mental state examining of the CAMHS patent to paediatric SHOs in order to improve their confidence in assessment.

**Methods:**

In order to assess initial confidence in assessing the mental-state of a CAMHS patient, a pre-teaching questionnaire was given to the paediatric SHOs. A 30-minute teaching session on the mental state exam was then carried out and a post-teaching questionnaire was then given to the same trainees.

**Results:**

Paired sample Wilcoxons signed rank test found that training significant improved trainees’ confidence in taking a psychiatric mental state exam (p= 0.005, r = 0.628), and improved their confidence in presenting a mental state exam (p = 0.0041, r = 0.6420).

**Conclusions:**

Being able to confidently assess the mental state of a CAMHS patient in an on call shift is important for the initial assessing paediatric trainee. However this is often not taught in the paediatric curriculum and trainees have expressed some anxiety in performing this assessment overnight, before a more comprehensive assessment by a CAMHS professional. A simple teaching session may help to reduce this anxiety and improve trainees’ confidence.

